# The Impact of Aging on Adipose Function and Adipokine Synthesis

**DOI:** 10.3389/fendo.2019.00137

**Published:** 2019-03-11

**Authors:** Peter Mancuso, Benjamin Bouchard

**Affiliations:** ^1^Department of Nutritional Sciences, School of Public Health, University of Michigan, Ann Arbor, MI, United States; ^2^Graduate Program in Immunology, School of Public Health, University of Michigan, Ann Arbor, MI, United States; ^3^Department of Epidemiology, School of Public Health, University of Michigan, Ann Arbor, MI, United States

**Keywords:** adipose tissue, adipokines, aging, menopause, cardiovascular disease, diabetes

## Abstract

During the last 40 years, there has been a world-wide increase in both the prevalence of obesity and an increase in the number of persons over the age of 60 due to a decline in deaths from infectious disease and the nutrition transition in low and middle income nations. While the increase in the elderly population indicates improvements in global public health, this population may experience a diminished quality of life due to the negative impacts of obesity on age-associated inflammation. Aging alters adipose tissue composition and function resulting in insulin resistance and ectopic lipid storage. A reduction in brown adipose tissue activity, declining sex hormones levels, and abdominal adipose tissue expansion occur with advancing years through the redistribution of lipids from the subcutaneous to the visceral fat compartment. These changes in adipose tissue function and distribution influence the secretion of adipose tissue derived hormones, or adipokines, that promote a chronic state of low-grade systemic inflammation. Ultimately, obesity accelerates aging by enhancing inflammation and increasing the risk of age-associated diseases. The focus of this review is the impact of aging on adipose tissue distribution and function and how these effects influence the elaboration of pro and anti-inflammatory adipokines.

## Introduction

The global population of individuals aged 60 years and older is expected to nearly double from 12 to 22% between 2015 and 2050 (1). Simultaneously, there has been a dramatic increase in the prevalence of obesity worldwide among developed and, more recently, low and middle income nations (2). Obesity exacerbates aging-associated inflammation by impairing insulin responsiveness and contributes to the pathophysiology of diseases frequently observed in the elderly (3). While increased weight and adiposity accompany aging, the redistribution of adipose tissue to the abdominal compartment is of greater concern. These changes occur for a number of reasons including declines in testosterone in men and estrogen in women following menopause, and alterations in the cellularity and function of subcutaneous adipose tissue (4, 5). Brown adipose tissue activity declines with age potentially as a result of reduced sympathetic nerve output and age-induced upregulation of the transcription factor FOXOA3 (6). In addition, the shift in the deposition of lipids to the abdominal adipose tissue compartment is associated with an increased risk of chronic disease (7). The ability of adipocytes to buffer dietary lipids declines with age and lipids are deposited in the liver and muscle which contributes to a low-grade state of inflammation, insulin resistance, and metabolic syndrome. Collectively, these changes in adipose tissue function and distribution during aging affect the synthesis of adipose tissue-derived mediators, or adipokines, known to regulate many physiologic processes including inflammation. This review will briefly describe global population trends, age-associated inflammation, and changes in adipose tissue function and distribution in aging and obesity, and discuss how these factors influence the production of pro and anti-inflammatory adipokines.

## An Increase in the Obese Elderly Population

The number of individuals aged 65 years and older is increasing to a point where 20% of the population in the US will be 65 years or older by 2030 (1). In addition, successful public health measures have reduced the number of deaths from infectious disease in low and middle income nations raising the number of individuals who are over the age of 60 years on a global scale. Unfortunately, a transition of nutrition, where western style diets rich in calories from fat and simple carbohydrates have replaced traditional diets across the globe increasing the prevalence of obesity, defined as having a body mass index (BMI) of ≥30. This has coincided with an increase in chronic illnesses known to be caused by excess adiposity (7). Weight increases with age and BMI peaks occur in people aged 50–59 years and adipose tissue reaches its peak between the ages of 60 and 79 years. In total, 38.5% of persons aged 60 and older in the US were obese (8, 9). The increased prevalence of global obesity appears to have been caused by the over consumption of highly-palatable, energy dense food, and a decline in energy expenditure as a consequence of sedentary behavior (10, 11). Increased life expectancy has the potential to improve quality of life in countries with growing elderly populations. However, if life extension is associated with excess adipose tissue and altered metabolic homeostasis, the added years of life may result in diminished health status as a consequence of age-associated chronic disease, loss of physical function, and frailty (12).

## Inflammation in Aging and Obesity

The term “Inflammaging” was originally coined by Claudio Franceschi to describe the chronic low-grade inflammation in the absence of infection driven by endogenous signals that accompany aging (3). In this scenario, the innate immune system is activated by the accumulation of cellular damage caused by reactive oxygen species (13–15). This inflammatory state increases the risk of cardiovascular disease, type 2 diabetes, arthritis, and several other ailments that compromise quality of life in the elderly (16–19). Likewise, a chronic state of low grade inflammation is observed in subjects with excess adiposity. Under these conditions, inflammation is initiated by the inability of adipose tissue to buffer dietary lipids resulting in lipotoxicity mediated by the ectopic deposition of lipids in the liver and skeletal muscle (20). Lipotoxicity in these tissues increases reactive oxygen species and activates serine threonine kinases such as c-jun N-terminal kinase (JNK), IκB kinase (IKK), and protein kinase C (PKC). These events disrupt insulin receptor signaling cascades and promote insulin resistance (15). In addition, bioactive lipid metabolites, diacylglycerol and ceramides, accumulate and negatively impact mitochondrial function, and biogenesis (21–23). These events are associated with the development of hepatic steatosis and muscle dysfunction and may trigger the development of sarcopenia (21–23). Inflammaging may also have a significant impact on the distribution and function of adipose as mentioned below.

## Adipose Tissue Depot Function and Distribution

The major adipose tissue depots include the visceral, subcutaneous, bone marrow, and perivascular compartments. It is becoming increasingly clear that these depots have distinctly different functions. For example, the visceral adipose tissue depot buffers dietary lipids by storing excess calories in the form of triglycerides (20). It releases this stored energy in response to physical activity and caloric deficits in order to provide fuel for physiologic functions in the post-prandial state and during fasting. The subcutaneous compartment provides insulation, cushioning, and serves as a long-term energy storage depot (7). The function of bone marrow adipose tissue is poorly understood but this tissue replaces hematopoietic cells during aging and is the most abundant source of adiponectin in mice and humans (24). Perivascular adipose tissue, that surrounds major and small arteries and veins, regulates thermogenesis and vascular tone (25). Brown adipose tissue is closely associated with the cervical, supraclavicular, and superior mediastinal vasculature in humans (26). The deposition of lipids in various adipose tissue depots is governed by sex hormones, the location of sex hormone receptors, catecholamines, and the activity of adipose triglyceride, hormone sensitive, and lipoprotein lipases (27).

## Sex Differences in Adipose Tissue Deposition With Age

Men have a lower percentage of body fat than women and tend to deposit more adipose tissue above the waist in abdominal visceral and subcutaneous compartments compared with pre-menopausal women. While visceral adipose tissue accounts for only 6–20% of total body fat, accumulation of fat in this depot is associated with an increased risk of metabolic syndrome, and cardiovascular disease (27). This is the distinguishing characteristic of the android pattern of adipose tissue deposition which is due to differences in the levels of sex hormones, testosterone and estrogen, and the adipose tissue depot specific expression of their receptors (27). In general, adipose tissue mass increases with age in response to a chronic positive calorie balance, reduced physical activity, and a lower basal metabolic rate (28). As men age, the increase in fat mass occurs predominantly above the waist with the expansion of the abdominal visceral and subcutaneous compartments and this has been attributed to declining levels of testosterone (29) Testosterone levels peak in men during puberty, begin declining by 1% annually between the ages of 20 and 30, and reach their nadir after the age of 70 (27). In addition to a decline in testosterone synthesis, physiologically available testosterone, free testosterone and testosterone bound to albumin, also declines as a consequence of increased levels of steroid hormone binding globulin (SHBG) which binds testosterone and prevents its contribution to adipocyte fat metabolism (29). While the mechanism by which testosterone affects adipose tissue deposition has not been clearly defined, studies conducted on adipocytes obtained from human visceral adipose tissue have demonstrated that testosterone enhances lipolysis and inhibits lipid incorporation (27).

In premenopausal women, adipose tissue is distributed predominantly in the gluteal femoral subcutaneous compartment and this is associated with a lower risk of cardiovascular disease compared with abdominal fat deposition (30). This is due to estrogen receptor alpha (ERα) expression in subcutaneous gluteal femoral adipose tissue depots which mediates lipoprotein lipase activity and triacylglycerol accumulation in adipocytes this region (27). After reaching menopause, estrogen levels decline in women, and the androgen to estrogen ratio increases. Consequently, there is a redistribution of lipids to visceral adipose tissue compartment and an increased risk of cardiovascular disease, hypertension, and diabetes (5, 27, 31). The androgen to estrogen ratio is also elevated in premenopausal women with polycystic ovarian syndrome (PCOS) (32). In this a condition, lipid redistribution is also evident resulting in increased abdominal visceral adiposity and an increased risk of cardiometabolic disease (32). In addition to changes in white adipose tissue with age, a decline in brown tissue activity in older adults has also been reported (24, 33, 34).

## Impact of Aging on Brown Adipose Tissue Activity

A notable change in adipose tissue distribution associated with aging and obesity is the loss of brown adipose tissue whose function declines with advancing years and increasing body fat percentage (26). Energy is released in the form of heat from lipids stored within brown adipose tissue with the upregulation of uncoupling protein-1 (UCP-1) (26). One potential mechanism behind the loss of brown adipose tissue involves the transcription factor forkhead box protein A3 (FOXA3) which increases with aging and visceral obesity (6). Ablation of FOXA3 protects against the development of obesity and insulin resistance in aged mice on a high fat diet and improves lifespan (6). Another proposed mechanism associated with a decline in brown adipose tissue with aging is a reduction in sympathetic drive (34). Brown adipose tissue is activated and recruited to generate heat by the sympathetic nervous system. In a study by Bahler et al. sympathetic nerve activity and brown adipose tissue recruitment and activity were lower in lean older men 50–60 years old vs. lean young men aged 20–28 years (34). Finally, age may affect brown adipose tissue adipokines produced by this tissue that are known to regulate precursor cell adipocyte commitment, differentiation, and factors that promote thermogenesis (35). Brown adipose tissue adipokines have been reviewed extensively by Villarroya et al. (35).

## Changes in Adipose Tissue Cellular Composition and Distribution with Aging

Adipose tissue is composed of mature adipocytes, preadipocytes, mesenchymal cells, and various cell types that make up the stromal vascular fraction including vascular endothelial cells, smooth muscle cells, fibroblasts, and several different types of immune cells (36–40). Mature adipocytes store excess calories in the form of triacylglycerol within vacuoles to provide energy to the host in times of a negative energy balance. During weight gain, adipose tissue expands with an increase in both number of adipocytes (hyperplasia) and volume (hypertrophy). The expansion of adipocytes by hyperplasia is associated with insulin sensitivity and metabolic control, which are characteristics of subcutaneous adipocytes. In contrast, adipose tissue expansion by hypertrophy is associated with reduced triacylglycerol storage capacity, ectopic lipid deposition, and impaired insulin sensitivity (20). It also leads to adipocyte necrosis, polarization of adipose tissue macrophages that assume a classically activated or M1 phenotype, and recruitment of additional monocytes and other immune cells from the circulation in response to the elaboration of chemokines such as CCL2 and CXCL5 (41, 42). Aging has a significant impact on the lipid storage capacity and the distribution of adipose tissue in human subjects. As mentioned above, body fat percentage increases with age mostly due to increases in visceral adipose tissue expansion (43). While this is primarily due to a chronic positive energy balance, it is also influenced by a shift in lipid storage from the subcutaneous to the visceral fat depot (43). The decline in subcutaneous fat depot storage and function is thought to occur through the decline in progenitor cell function and the accumulation of senescent adipose tissue cells (44). Mesenchymal cells are progenitor cells found within the stromal vascular fraction that can undergo differentiation into preadipocytes and eventually mature adipocytes. The progenitor cell populations isolated from aged adipose tissue have reduced function and an impaired ability to incorporate lipids and potential to differentiate into preadipocytes (45, 46). In addition, there is an accumulation of senescent cells that lack the ability to divide in response to metabolic stress (47). These senescent cells express a distinguishing set of markers such as p16, p21, caveolin-1, and senescence-associated β-galactosidase (SA-β-gal). The secretion of bioactive mediators produced by these cells, referred to as having a senescent-associated secretory phenotype, is characterized by an increase in IL-6 and plasminogen activator inhibitor (PAI-1) (48, 49). Other factors that contribute to cellular senescence include telomere shortening and mitochondrial dysfunction (50, 51).

## Effect of Aging on Responsiveness to Autonomic Nerve Function

Aging is associated with a decline in autonomic nervous system function which diminishes the ability of the elderly to respond to environmental and internal stimuli (52). These impairments in autonomic system function include the loss of some autonomic nerve projections, alterations in the output and balance of sympathetic and parasympathetic outflow to visceral organs, and reduced receptor responsiveness (52). One notable example is the impact of aging on catecholamine-induced lipolysis in visceral adipose tissue (53). Under normal metabolic controls, norepinephrine released by sympathetic nerves induces lipolysis of triglycerides stored in adipocytes residing in visceral adipose tissue. Norepinephrine is metabolized by the enzyme monoamine oxidase A which is expressed in adipose tissue and sympathetic neuron-associated macrophages. In aged mice, adipose tissue macrophages are recruited to expanding visceral adipose tissue and activated in a NLRP3-inflammasome-dependent manner resulting in an increase in monoamine oxidase A and norepinephrine degradation (53). In human adipose tissue from aged humans, the import and degradation of norepinephrine is enhanced in sympathetic neuron-associated macrophages. In this circumstance, the expression of a sodium-dependent norepinephrine transporters (SLA6A2) and monoamine oxidase A are increased with aging resulting in greater clearance of norepinephrine and reduced lipolysis in visceral adipocytes (54). These changes are associated with an expansion of visceral adipose tissue, impaired insulin sensitivity, and a decline in subcutaneous adipocytes number and function with age (43, 55, 56). Ultimately, adipose tissue endocrine function and adipokine secretion are impacted as discussed below in section Effect of Aging on Adipose Tissue Adipokine Secretion.

## Effects of Aging on Adipose Tissue Adipokine Secretion

Adipose tissue is the largest endocrine gland in the human body that secretes hundreds of bioactive molecules. Among these hormones are the adipokines, proteins secreted by adipocytes and stromal vascular cells that have profound effects on several physiologic functions including appetite and satiety, adipogenesis, reproduction, glucose homeostasis, energy expenditure, inflammation, and several other physiologic functions (57). The impact of aging on adipose tissue adipokine secretion is influenced by age associated changes in adipose tissue distribution, cellular composition, local tissue inflammation, sex hormones, and cellular differentiation (43, 58–61). These combined effects alter the balance of local and systemic pro and anti-inflammatory adipokine levels. In general, the expansion of visceral adipose tissue by hypertrophy is associated with an increase in proinflammatory adipokines and a decline in anti-inflammatory mediators (42). With aging, nearly all adipokine levels are elevated in comparison with younger individuals with the same body fat percentage as mentioned below.

### Pro-Inflammatory Adipokines

#### Leptin

Leptin is a proinflammatory adipokine best known for its role in appetite, satiety, and energy expenditure (62–64). Leptin is produced by adipose tissue and circulates in blood in proportion to total fat mass. It informs the central nervous system about the status of peripheral energy storage and contributes to the defense against weight loss. For example, when adipose tissue levels decline with weight loss, circulating leptin declines and this reduces the amount of leptin that reaches the hypothalamic nuclei in the brain that controls energy homeostasis. In response to lower leptin, appetite increases which promotes feeding. As energy intake increases, adipose tissue lipid levels rise and this restores circulating leptin and diminishes appetite to pre-weight loss levels (65). Obesity is a state of excess adipose tissue where elevated leptin levels fail to reduce appetite and increase energy expenditure. The failure of leptin to restore metabolic homeostasis in obesity is described as state of leptin resistance. Obesity induces leptin receptor induced inhibitory signals, hypothalamic inflammatory stimuli, endoplasmic reticulum stress, and gliosis. Collectively, these events promote leptin resistance in obesity (65).

In general, levels of leptin are higher in women compared with men and this difference is not only due to a higher percentage of body fat in women but is also affected by androgens (66, 67). The rate of leptin production per unit mass of adipose tissue is higher in women vs. men and this difference can be attributed to testosterone which suppresses leptin synthesis (67). Interestingly, higher leptin synthesis has been reported in subcutaneous adipose tissue compared with that observed in omental fat in overweight and obese humans (68). Despite the decline in subcutaneous fat observed in older individuals, leptin is correlated with total fat mass throughout the life course and age does not have an independent effect on leptin and adiposity in men or women (43, 61, 69–71). Therefore, the increased levels of circulating leptin in older adults is primarily due to increased fat mass in comparison with younger adults. In addition, It has been hypothesized that leptin responsiveness may be diminished with increasing age due to impaired hypothalamic leptin receptor signaling which has been demonstrated in aged rats (72, 73). While the mechanism responsible for age related leptin resistance in humans has not been demonstrated, reduced expression of the short form of the leptin receptor (LepRa) in peripheral blood monocytes has been reported in aged humans. LepRa is known to transport leptin across the blood brain barrier (71). Whether age diminishes hypothalamic leptin responsiveness in humans remains to be seen that is linearly correlated with total body fat and BMI.

#### Resistin

Another proinflammatory adipokine that is known to increase with obesity is resistin which was originally described by Steppan et al. as mediating insulin resistance in mice (74). Research on the role of resistin in human disease associated with obesity has been challenging due to differences between mouse and human resistin in homology and cellular sources. For example, in mice, resistin is produced by adipose tissue and monocytes. In humans, monocytes and macrophages but not adipose tissue, produce this adipokine. Resistin has been implicated as an important proinflammatory mediator in atherosclerosis since it induces monocyte-endothelial cell interactions by increasing the expression of intracellular adhesion molecule-1 (ICAM-1) and vascular endothelial adhesion molecule-1 (VCAM-1) (75–77). While age does not appear to affect resistin levels independent of fat mass, elevated levels of this adipokine are associated with an increased risk of cardiovascular disease in elderly men and women and insulin resistance in patients with a history of coronary intervention (70, 76, 78, 79).

#### Chemerin

Chemerin is a hormone secreted by adipose tissue that activates the chemokine-like receptor-1 (CMKLR-1) to initiate innate and adaptive immune responses (80). It is a secreted prohormone that requires further processing by proteases in order to become biologically active (81). This proinflammatory adipokine acts as a chemoattractant for immature dendritic cells, macrophages, and natural killer cells that express CMKLR-1 (82). It is correlated with BMI and elevated in individuals with central obesity and may be an important link between excess adiposity and type 2 diabetes (81, 83, 84). It promotes the secretion of adipokines that induce insulin resistance in diabetes. Chemerin was positively associated with age but it is not clear if increased chemerin occurs as a consequence of aging or the accumulation of visceral adipose tissue with advancing years (80). In addition, there is uncertainty about a specific role of chemerin in metabolic diseases associated with excess adiposity since weight loss and improved metabolic control are associated with reduced chemerin levels. This might suggest that chemerin synthesis is responsive to metabolic status rather than it being a bioactive mediator that promotes inflammation and insulin resistance independent of other proinflammatory mediators (85). Evidence of sexual dimorphism for this adipokine is supported by increased levels of chemerin mRNA in subcutaneous vs. visceral adipose tissue compartments in women in a report by Alfadda et al. (86). In men and women with polycystic ovarian disease, a condition characterized by elevated levels of testosterone and increased visceral adipose tissue, mRNA levels of chemerin were elevated in the visceral compared with subcutaneous adipose tissue compartments (87).

#### Retinol Binding Protein 4 (RBP4)

RBP4 is member of the lipocalin family of proteins that binds retinoic acid and transports it to peripheral tissues and whose expression increases with BMI, total body fat, and hepatic adipose tissue (88, 89). In addition to adipocytes, it can be produced by the liver and macrophages. RBP4 may directly promote adipose tissue inflammation and insulin resistance in humans since enhanced expression of RBP4 in transgenic mice results in adipose tissue inflammation and macrophage accumulation (90). In addition, RBP4 expression is associated with the percentage of trunk fat (central adiposity) and insulin resistance in young but not elderly subjects (91). Interestingly, RBP4 levels are significantly elevated in aged individuals independent of central adiposity (91). Circulating levels of RBP4 are higher in male compared with female mice and humans (92, 93).

#### Lipocalin 2 (LCN2)

LCN2, also referred to as neutrophil gelatinase-associated lipocalin, is another member of the lipocalin family of proteins that transports lipid molecules such as retinoic acid, arachidonic acid, leukotriene B_4_, and platelet activating factor in circulation (94). It is produced by adipocytes at high levels in mice and humans in response to inflammatory stimuli and the impact of age on this proinflammatory adipokine is unknown (95, 96). However, adipose tissue-derived LCN2 has been shown to promote the pathogenesis of renal injury, a condition that is more prevalent in aged individuals with type 2 diabetes (97, 98). In addition, it may also play an important proinflammatory role in adipose tissue remodeling during visceral fat expansion (99). Since LCN2 is from the same family of proteins as RBP4, a lipid transporter whose synthesis increases with advancing years, age may affect the expression of LCN2 and influence the progression of diseases associated with obesity (91). Like chemerin, a sexual dimorphic pattern of LCN2 mRNA expression has been observed in humans with higher levels in visceral vs. subcutaneous adipose tissue depots in men and women with polycystic ovarian disease. In women, LCN2 transcripts are higher in the subcutaneous vs. visceral compartments (87).

#### Classical Proinflammatory Cytokines CCL2, IL-1β, IL-6, IL-12, IL-18, and TNF-α

Age associated changes to adipose tissue increase the synthesis of classical cytokines (100–104). As noted early, aging results in the redistribution of lipids that accumulate in visceral adipose tissue (43). This results in an increase in adipocyte hypertrophy since fat mass expansion via adipocyte hyperplasia is inhibited by an age-related decline in the ability of progenitor cells to differentiate into preadipocytes. Proinflammatory cytokines also inhibit preadipocyte differentiation and maturation, and promote adipocyte senescence (44–46, 56, 60). Proinflammatory cytokines (IL-1β, IL-6, TNF-α) secreted by adipose tissue macrophages reduce PPAR-γ expression, an important transcription factor that induces adipogenesis (42). Monocytes are recruited to visceral adipose tissue in response to chemokines such as CCL2 and these cells differentiate into adipose tissue macrophages (105). These proinflammatory mediators, associated with the M1 classically activated macrophage phenotype, impair insulin sensitivity and glucose tolerance (42, 106, 107). An increase in the adipose tissue population of CD8+ T cells and a decline in regulatory T cells are thought to contribute to the promotion and maintenance of the M1 phenotype of adipose tissue macrophages with aging (107–109). In general, proinflammatory adipokines increase with age due to either increased adipose tissue mass or an enhancement of inflammation that promotes increased synthesis. In opposition to these proinflammatory bioactive molecules are the anti-inflammatory adipokines which also have been observed to increase with age. The overabundance of proinflammatory adipokines associated with excess central adiposity may outweigh the effects of the anti-inflammatory adipokines mentioned below.

### Anti-inflammatory Adipokines

#### Adiponectin

The anti-inflammatory adipokine, adiponectin, is the most abundantly expressed adipokine found in human serum at levels in the μg/ml range (110). In contrast to all other adipokines, it is predominantly produced by bone marrow adipose tissue (24). Adiponectin forms complex aggregates that circulate in high (HMW), medium, and low-molecular weight forms with the HMW form having the greatest effect on improving insulin sensitivity and glucose tolerance (111). There are two isoforms of the adiponectin receptor (AdipoR1 and AdipoR2) that are expressed in vascular endothelial cells, monocytes and macrophages, skeletal and cardiac muscles cells, and adipocytes (60, 112). Adiponectin plays a protective role against cardiovascular disease since it inhibits foam cell formation, adhesion molecule expression, and endothelial cell-monocyte interactions (113, 114). It also inhibits the synthesis of proinflammatory cytokines such as IL-6, IL-18, and TNF-α synthesis by blocking NF-κB activation (115, 116). Adiponectin promotes adipogenesis and the expansion of adipose tissue via hyperplasia, a mechanism of fat pad expansion that reduces adipose tissue inflammation and maintains insulin responsiveness and glucose homeostasis (117). Peroxisome proliferator-activated receptor-gamma (PPARγ) agonists, such as the glitazone drugs, increase adiponectin synthesis (118, 119). Serum adiponectin levels are elevated with age, fasting, treatment with glucocorticoids, and conditions that enhance the expansion of bone marrow adipose tissue (24, 120–123). In contrast, lower levels of adiponectin are associated with obesity, cigarette smoking, and oxidative stress (124, 125). Centenarians have higher levels of adiponectin and this may be associated with longevity (126, 127). While elevated adiponectin may be associated with improved metabolic status in the elderly, it has also been associated with reduced physical functioning (127, 128). Serum adiponectin levels are higher in women than in men (129).

#### Vaspin

Visceral adipose tissue-derived serpin (Vaspin), a member of the serine protease inhibitor family of proteins, is expressed by visceral fat in rats and humans (130). It was originally found in Otsuka Long-Evans Tokushima rats and associated with obesity and insulin sensitivity in rats and humans (130, 131). Higher levels of vaspin have been reported in women vs. men (132). Exogenous administration of vaspin improves insulin responsiveness and glucose tolerance in mice (132). In addition, vaspin levels increase following aerobic exercise in untrained individuals (132, 133). In addition to adipose tissue, vaspin is produced by the β-cells of the pancreas, skin, and the hypothalamus in mice (133). Vaspin declines with aging and insulin sensitivity but increases following treatment with insulin or pioglitazone (130, 133). Interestingly, vaspin mRNA is undetectable in the adipose tissue of lean adults (BMI < 25) but increases in visceral and subcutaneous adipose tissue of individuals in association with BMI, body fat percentage, and insulin sensitivity (134).

#### Secreted-Frizzled-Related Protein 5 (SFRP5)

SFRP5 is an anti-inflammatory and insulin sensitizing adipokine that promotes adipogenesis by inhibiting wingless type MMTV integration site (Wnt) 5a/JUN N-terminal kinase (JNK) intracellular signaling events in macrophages and preadipocytes suppressing the synthesis of TNF-α‘, IL-1β, and CCL2 (60, 135, 136). Its production in adipose tissue promotes adipose tissue expansion via hyperplasia (42). Levels of SFRP5 are lower in individuals with obesity, diabetes, non-alcoholic fatty liver disease, and hypertension and negatively correlated with C-reactive protein (CRP) (60, 137–142). SFRP5 levels increase with age and are higher in female compared with males in both rodents and humans (143).

#### Omentin-1

Omentin-1 is an anti-inflammatory adipokine that is expressed in omental and epicardial fat (visceral adipose compartment) as wells as bronchial goblet cells, mesothelial cells, vascular cells, Paneth cells within the small intestine, colon, and ovaries (144). While the isoform omentin-2, has been identified, its distinct biologic function is unknown. Although the receptor and physiological functions of omentin-1 are unknown, it signals through AMP-kinase/AKT/NF-κB/MAP Kinase (ERK, JNK, p38) pathways. In general, lower levels of omentin-1 are associated with systemic inflammation and impaired metabolic control such as in obesity, type I and type 2 diabetes, coronary artery disease, metabolic syndrome, and hepatic steatosis (144–149). Omentin-1 levels increase with age, weight loss, olive oil rich diets, aerobic exercise, administration of fibroblast growth factor (FGF)-21, and following treatment with drugs used to improve insulin responsiveness (144, 150–156). Omentin-1 may be a promising treatment for atherosclerosis since exogenous administration of this adipokine prevents atherosclerosis in Apo-e deficient mice by reducing reactive oxygen species synthesis, suppression of TNF-α—induced intracellular adhesion molecule (ICAM) and vascular endothelial cell adhesion molecule (VCAM) expression, and monocyte interaction with vascular endothelium (157).

#### C1q/TNF-Related Proteins (CTRPs)

CTRPs are anti-inflammatory adipokines that are structurally similar to adiponectin and 15 different isoforms have been identified (158). CTRPs activate intracellular signaling events via AMP-kinase which inhibits proinflammatory cytokine production (159). CTRPS enhance insulin responsiveness and glucose tolerance after high intensity interval training (160). CTRP1, CTRP9, and CTRP12 increase with insulin sensitivity and promote glucose uptake (161–163). CTRP1 is somewhat different than other CTRP family members since it is produced by non-adipocytes within the stromal vascular fraction of adipose tissue and increases with obesity and hypertension (158). CTRP1 is associated with atherosclerosis and promotes monocyte-endothelial cell interactions (164). In contrast, CTRP3 has potent anti-inflammatory effects since it blocks LPS–TLR4 mediated inflammation (165). CTRP3 is lower in patients with type 2 diabetes and its levels are inversely proportional to blood glucose and insulin (166). Interestingly, serum levels of CTRP3 and CTRP5 increase following 8 weeks of aerobic training in middle-aged and older men and women and this was associated with reduced arterial stiffness (167). In liver cells, CTRP13 improves glucose uptake and insulin resistance in lipid laden hepatocytes (168). Finally, CTRP11 and CTRP14 have been shown to stimulate angiogenesis of endothelial cells and these CTRP isoforms may be important in adipose tissue vascularization (169). More research is needed to study the potential use of CTRPs known to improve insulin sensitivity and glucose tolerance in patients with type 2 diabetes.

## Conclusions

**Figure 1:**
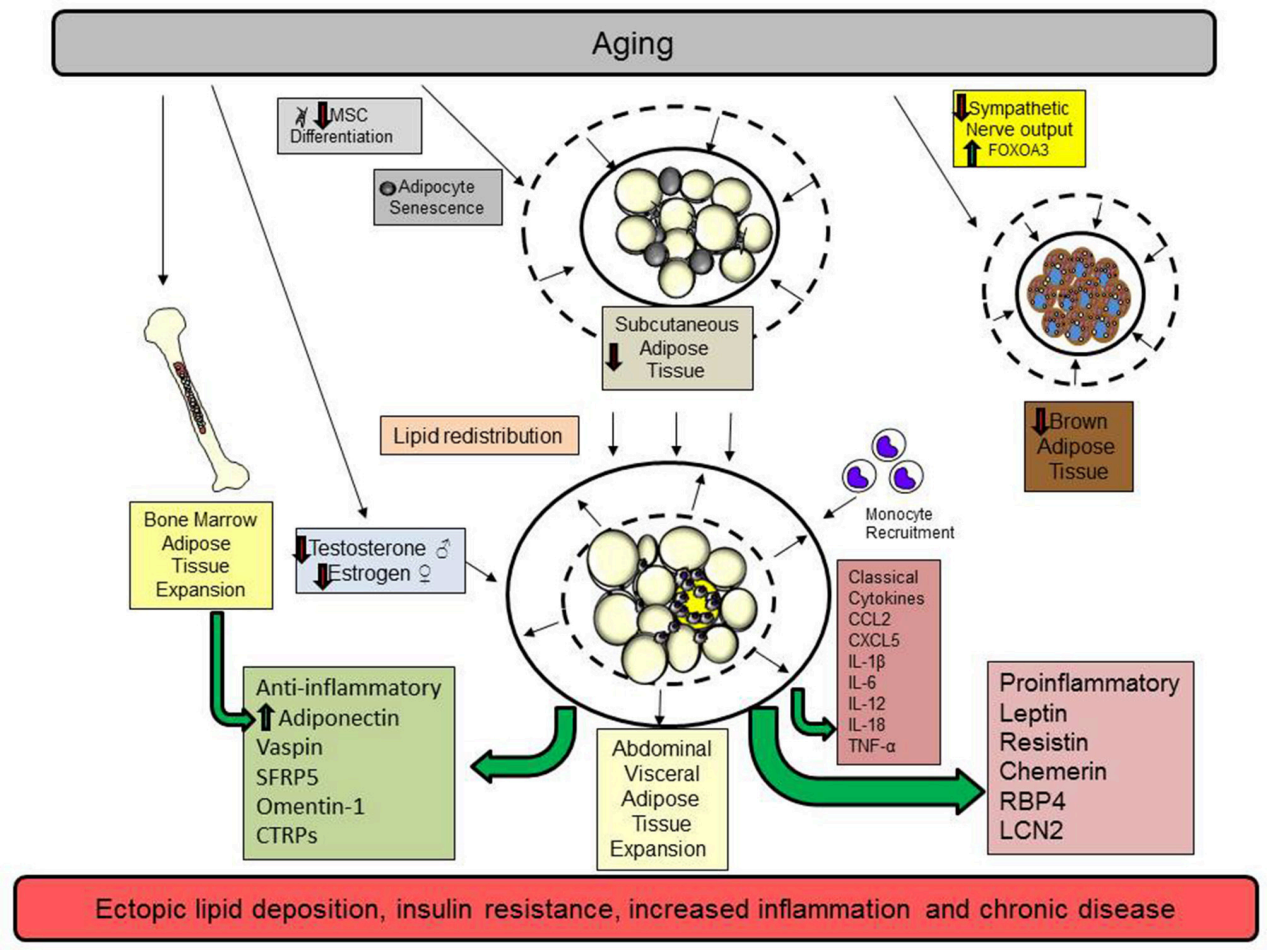
Aging promotes the redistribution of lipids from the subcutaneous to the abdominal visceral compartment. Aging promotes cellular senescence and impairs mesenchymal stem cell (MSC) differentiation in subcutaneous adipose tissue. These changes diminish adipocyte function by reducing preadipocyte maturation, restrict adipocyte hyperplasia, and reduce subcutaneous adipocyte mass. In addition, declining sex hormones in men (testosterone) and women (estrogen) also contribute to visceral adipose tissue expansion. Brown adipose tissue declines because of reduced sympathetic output and increases in the transcription factor FOXOA3. Subsequently, lipids are redistributed to the abdominal adipose tissue depot. As adipose tissue expands in this compartment, adipocytes undergo hypertrophy, a process that contributes to adipocyte necrosis, adipose tissue inflammation, and the elaboration of proinflammatory classical cytokines and adipokines. Monocytes and other immune cells are recruited to the visceral adipose tissue depot to remove necrotic adipocytes and participate in tissue remodeling limiting lipid storage. Ultimately, these events contribute to ectopic lipid storage and insulin resistance. Bone marrow adipose tissue expands to replace hematopoietic cells and this is associated with increased adiponectin synthesis. While many pro and anti-inflammatory adipokines increase with age, the dominance of proinflammatory adipokines shifts the balance to favor a chronic state of inflammation.

There has been a dramatic increase in the number of people over the age of 60 years globally. Unfortunately, these extra years may be associated with a lower quality of life due to chronic illness and metabolic disease associated with obesity. Aging promotes the redistribution of lipids from the subcutaneous to the abdominal visceral compartment. The process is summarized in Figure 1. The inflammation that occurs in aging is exacerbated by excess adiposity contributing to an increased risk of type 2 diabetes, cardiovascular disease, and many other diseases associated with obesity. To counter these events, interventions that maintain adipose tissue function during aging, such as eliminating senescent cells, exercise, weight loss, and drugs that promote insulin sensitivity, may increase life expectancy and ultimately, quality of life. More research is needed to assess the impact of sex differences and aging on adipokine synthesis and function and whether these differences contribute to or are a consequence of diseases associated with aging.

## Author Contributions

PM wrote and edited the manuscript. BB helped find references, edited manuscript, and provided expertise in adipokine assessment.

### Conflict of Interest Statement

The authors declare that the research was conducted in the absence of any commercial or financial relationships that could be construed as a potential conflict of interest.
